# Motor control exercise program versus standard care in the treatment of lumbopelvic pain in pregnant women: a randomized controlled pilot trial

**DOI:** 10.1186/s12998-026-00655-x

**Published:** 2026-05-30

**Authors:** Catherine Daneau, Véronique Babineau, Andrée-Anne Marchand, André Bussières, Julie O’Shaughnessy, Martin Descarreaux, Stephanie-May Ruchat

**Affiliations:** 1 Department of Anatomy, Université du Québec à Trois-Rivières, 3351 Bd des Forges, Trois-Rivières, QC G8Z 4M3 Canada; 2 Department of Obstetrics and Gynaecology, Centre intégré universitaire de santé et de services sociaux de la Mauricie et- du-Centre-du-Québec, University of Montréal, Trois-Rivières, Canada; 3 Department of Chiropractic, Université du Québec à Trois-Rivières, 3351 Bd des Forges, Trois-Rivières, QC G8Z 4M3 Canada; 4 School of Physical and Occupational Therapy, McGill University, 845 Rue Sherbrooke O, Montréal, QC H3A 0G4 Canada; 5 Department of Human Kinetics, Université du Québec à Trois-Rivières, 3351 Bd des Forges, Trois-Rivières, QC G8Z 4M3 Canada

**Keywords:** Exercise, Motor control, Low back pain, Pelvic girdle pain, Pregnancy

## Abstract

**Background:**

Lumbopelvic pain is highly prevalent in pregnant women and although exercises are part of the strategies used to manage pregnancy-related lumbopelvic pain (PLBPP), available evidence regarding their effectiveness is inconclusive. The primary objective of this pilot study was to assess the feasibility of implementing a motor control exercise program for pregnant women presenting with a history of, or currently experiencing lumbopelvic pain. The secondary objective of the study was to explore the preliminary effectiveness of the program.

**Methods:**

Women aged 18–40 years with a singleton pregnancy at gestational age ≤ 20 were eligible if they had a history of lumbopelvic pain or were currently experiencing their first PLBPP episode. Participants were randomly allocated to an intervention group (*n* = 16) or a control group (*n* = 16). The intervention group received standard prenatal care and participated in three 40-minute exercise sessions per week (one supervised and two unsupervised) from gestational age ≤ 20 weeks through 34–36. The control group received only standard prenatal care. Primary outcomes were recruitment, retention, and adherence rates, safety and acceptability of the intervention, whereas secondary outcomes included PLBPP frequency and intensity, PLBPP-related disability and several other PLBPP-related outcomes. Descriptive statistics were used to assess the feasibility and the preliminary effectiveness of the intervention.

**Results:**

Over a 15-month period, 32 participants were recruited (April 2021 to July 2022). Of these, 26 (11 in the intervention group and 15 in the control group) attended the post-intervention visit, yielding an 81.3% retention rate. The adherence rates were similar for supervised and unsupervised exercise sessions; however, acceptability was higher for supervised sessions than for unsupervised sessions. No adverse events were reported. At pre-intervention, participants’ characteristics were similar between both groups except for education levels (higher in the control group). Preliminary results showed no clear differences in PLBPP frequency, intensity or associated disability between groups.

**Conclusions:**

The motor control exercise program appears safe and feasible, with minor modifications. Optimization of recruitment strategies and participant adherence is warranted before proceeding to a full-scale randomized controlled trial.

**Trial registration:**

The study has been registered at the US National Institutes of Health Clinical trials registry (Clinicaltrials.gov) (05/02/2020) NCT04253717.

**Supplementary Information:**

The online version contains supplementary material available at 10.1186/s12998-026-00655-x.

## Background

During pregnancy, around 60% of women suffer from lumbopelvic pain which is a pain located in the lumbar (low back pain, LBP) and pelvic (pelvic girdle pain, PGP) regions [1, 2]. Several risk factors for developing pregnancy-related lumbopelvic pain (PLBPP) have been identified, among which a history of LBP or PGP prior to pregnancy appears to significantly increase the risk of PLBPP [3]. Moreover, the presence of PLBPP has been shown to negatively affect participation in regular physical activity (PA) [4]. PA can be defined as any bodily movement produced by skeletal muscles that requires the expenditure of energy [5] and is considered an important component of a healthy pregnancy due to its many health benefits for the pregnant woman and their future child. The latest PA guidelines [6], including the 2019 Canadian guideline for PA throughout pregnancy [7] recommend that all pregnant women without contraindications participate in regular PA throughout pregnancy and aiming for at least 150 min of moderate-intensity activity per week across various modalities [7].

Exercise, a sub-category of PA defined as a planned, structured and repetitive activity with a specific goal [5], is one of the strategies used to prevent or limit physical pain, including LBP in the general population [8] but also in pregnant women. In a systematic review evaluating the effects of prenatal PA interventions on several pregnancy-related outcomes, the effectiveness of land-based exercise interventions in reducing both the prevalence and intensity of PLBPP was examined [9]. Ten studies were included, and the interventions included exercises to strengthen the abdominal, lumbar and pelvic regions as well as walking, stretching, relaxation, and breathing. The authors concluded that the effects of exercise interventions on PLBPP intensity were inconsistent, with only five studies out of 10 showing a significant positive effect [9]. Furthermore, the exercise interventions had no effect on the prevalence of PLBPP [9]. These mixed results might be explained, at least in part, by the different types of exercise used to manage PLBPP in these studies.

Stabilization exercises, also known as motor control exercises, have been shown to be effective for LBP management in different non-pregnant populations. The primary role of motor control exercises is to control intersegmental spinal movements while allowing patients to regain control and coordination of their spine and pelvis using motor learning principles [10], whereas the aim of conventional exercises is to improve basic physical abilities using large, global movements.

A systematic review focusing on non-pregnant individuals with persistent non-specific LBP showed that motor control exercises, alone or as a supplement to physical therapy focusing on specific spinal stabilizing exercises, were effective in reducing LBP intensity and disability [11]. Beyond pregnancy, evidence indicates that motor control exercise is an accepted option for chronic non‑specific LBP, with outcomes broadly comparable to other exercise forms, supporting a pragmatic selection based on clinician expertise, patient preference, feasibility, cost, and safety rather than presumed superiority [12]. Similarly, a recent systematic review, carried out among postpartum women, examined the impact of stabilizing exercises (abdominal, lumbar multifidus, pelvic floor muscles) on lumbopelvic pain intensity, disability and quality of life. The results showed a significant reduction in lumbopelvic pain intensity and disability after stabilizing exercises, but failed to show a significant improvement in quality of life [13]. In pregnant women, evidence regarding the effect of exercise on the incidence of PLBPP is mixed and characterized by heterogeneous exercise modalities; however, prenatal exercise has been shown to reduce pain severity compared with no exercise [14], which supports continued evaluation of structured exercise approaches such as motor control programs. This mechanism-based rationale (targeting deep trunk and pelvic stabilizers and lumbopelvic coordination) motivated our choice of a motor control approach [12].

At the time this motor control exercise intervention was proposed, some studies reported a positive impact of stabilizing exercises on pain intensity during pregnancy, but data were scarce [15]. More studies were thus needed to better document the impact of this type of exercise on PLBPP. Consistent with clinical guidance, exercise during pregnancy is considered safe and desirable in the absence of contraindications, reinforcing the suitability of testing a structured motor control exercise program in this population [16].

Therefore, the first objective of this pilot study was to assess the feasibility of implementing a motor control exercise program for pregnant women presenting a history of lumbopelvic pain or currently suffering from PLBPP by evaluating recruitment, retention, adherence rates as well as safety and acceptability of the intervention. The hypothesis was that the motor control exercise program will show adequate recruitment, high adherence, low attrition, and will be safe and acceptable among our population. The second objective was to explore the preliminary effectiveness of the motor control exercise program. The secondary hypothesis was that the proposed motor control exercise program will show exploratory indications of benefit (as trends toward reduced PLBPP frequency or intensity and reduced disability), acknowledging that the study was not powered to detect effectiveness.

## Methods

### Study design

This pilot study was a parallel randomized controlled trial involving pregnant women with either a history of lumbopelvic pain or current PLBPP. Participants were randomized to either the control (standard prenatal care) or intervention group (standard prenatal care combined with a motor control exercise program) with an allocation ratio of 1:1.

This study was approved by the institutional review boards of the Université du Québec à Trois-Rivières (CER-19-259-07.20) and the Centre Intégré Universitaire de Santé et de Services Sociaux de la Mauricie-et-du-Centre-du-Québec (CIUSSS-MCQ) (CÉRM-2019-004-01). It was registered at Clinicaltrials.gov (05/02/2020): NCT04253717. More details about the study have been published elsewhere [17]. All participants provided written informed consent after having received detailed oral and written information about the study prior to enrollment [17], in accordance with the certification delivered by the institutional review boards before any intervention. This pilot study was reported in accordance with the CONSORT extension for randomized pilot and feasibility trials [18], and the completed CONSORT checklist is provided as supplementary material. The appropriate citation has been included as: The reporting of the intervention followed the Template for Intervention Description and Replication (TIDieR) guidelines [19], which the corresponding checklist is also included as supplementary material. No changes to the methodology were made after the project began.

### Sample size

To assess the feasibility and to plan for a larger study, it was suggested to include 10 to 20 participants per group [20]. Forty pregnant women were therefore randomly allocated to the control (20 participants) or intervention (20 participants) group. Based on our previous cohort study in which we recruited 40 pregnant women in 12 months, with a retention rate of 80% [21], recruiting 40 women over a 12-month period was deemed feasible.

### Recruitment & eligibility criteria

Recruitment took place from April 2021 to July 2022. Participants were recruited at two local medical clinics, at the hospital department of gynecology and obstetrics, in the local community and via social media. The inclusion criteria were as follows: women aged 18–40 years, carrying a singleton pregnancy, ≤ 20 weeks’ gestation, and with either a history of lumbopelvic pain (current or previous) or a first episode of PLBPP of at least two weeks’ duration. The exclusion criteria were the following: rheumatic inflammatory disease, infectious disease, neuromuscular disease, vascular disease, connective tissue disease, severe disabling pain, and neurologic signs and symptoms. Women unable to understand or speak French, those unwilling to be randomized or presenting contraindications to exercise [7] were also excluded from the study. Women who met the inclusion criteria and were willing to participate were met at the university laboratory for the pre-intervention visit. During this visit, women completed several questionnaires to collect information about baseline characteristics, history of lumbopelvic pain, PA levels (Pregnancy Physical Activity Questionnaire, PPAQ [22]), functional disability (Pelvic Girdle Questionnaire, PGQ [23]), fear avoidance behavior (Tampa Scale of Kinesiophobia, TSK [24]), anxiety (State-Trait Anxiety Inventory, STAI-Y [25]) and depression (Beck Depression Inventory, BDI [26]). The presence of PLBPP was confirmed using different recommended clinical tests [2, 27] : Patrick’s FABERE test (pain on the medial side of the knee and femur or in the inguinal region [28, 29]), Posterior Pelvis Pain Provocation test (pain in the gluteal area on the provoked side [28–30]), Active Straight Leg Raise test (pain in the lumbar region [31, 32]), Trendelenburg (weakness observed by hip descent on the flexed side [28]) and Menell’s test (pain in the coxo-femoral joint [28]). LBPP was confirmed in women having at least one positive test.

### Randomization

At the end of the pre-intervention visit, participants were randomly allocated to the control or intervention group. The randomization sequence generation was performed by an independent research assistant using a computer random number generator. The allocation sequence concealment was performed using sequentially numbered, opaque and sealed envelopes. Two minimization criteria were considered to ensure good balance of factors known to affect the natural history of PLBPP: baseline PLBPP intensity (one point was attributed if PLBPP intensity was ≥ 10/100) and baseline PA levels (one point was attributed if total activity score was ≥ 300 on the Pregnancy Physical Activity Questionnaire). Participants were not blinded to intervention allocation, but the content of the exercise sessions was shared only with those allocated to the intervention group to prevent cross-contamination between groups. The kinesiologist who supervised the exercise sessions was not involved in the pre- and post-intervention evaluations. The team member who ran the pre- and post-intervention evaluations and who managed the database was not blinded to group allocation.

### Intervention

#### Intervention group

**Fig. 1:**
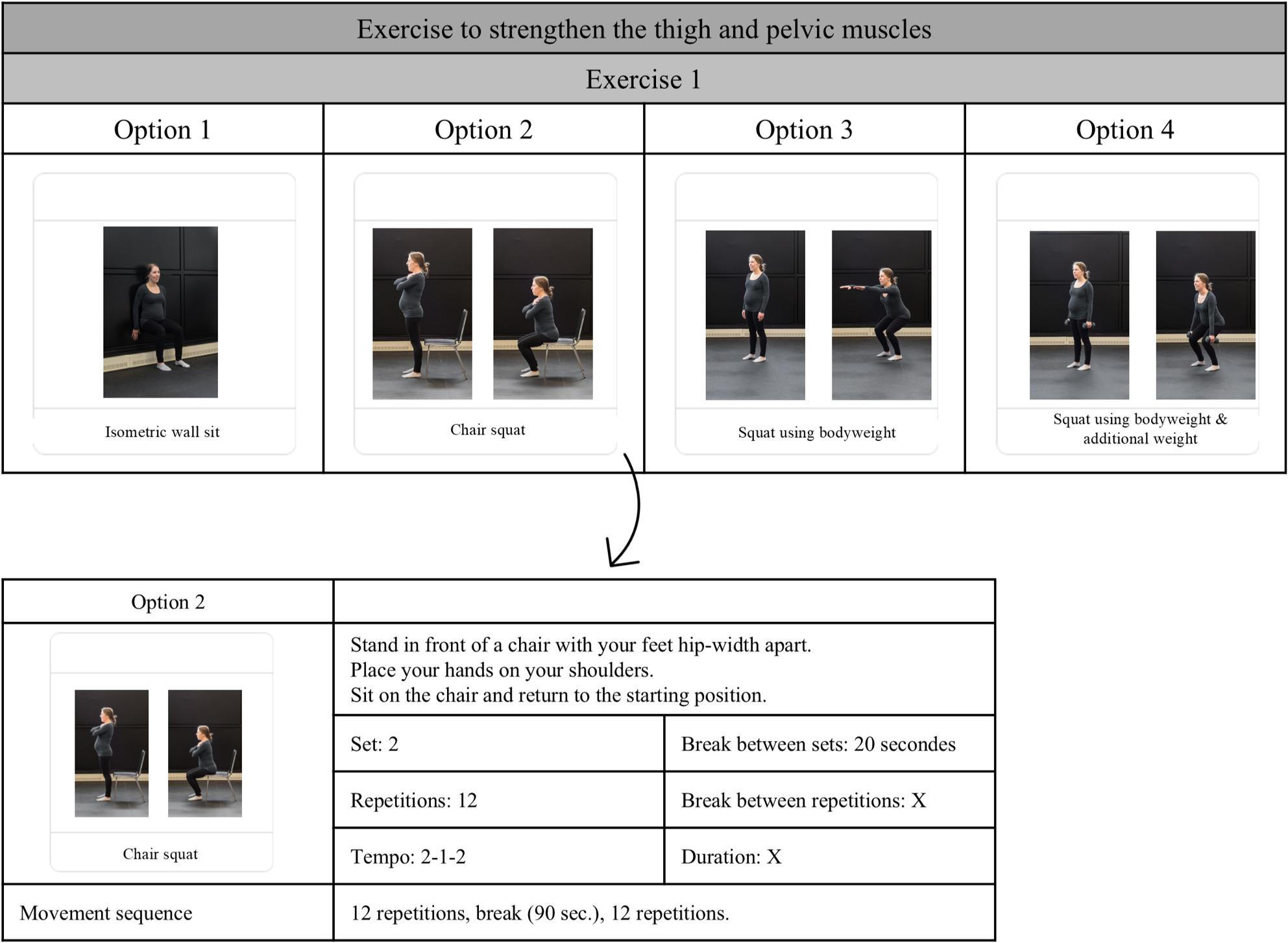
Examples of exercise options (with varying levels of difficulty) for strengthening the thigh and pelvic muscles. For each exercise, several information was included on the participant’s exercise program: technical points for performing the exercise, number of sets and repetitions, tempo or duration of the exercise, and the duration of the break between sets or repetitions

Women randomized to the intervention group received standard prenatal care, including basic information on what to do when suffering from PLBPP which is provided in the practical guide *From Tiny Tot to Toddler* [33], and participated in a motor control exercise program, consisting of three 40-minute exercise sessions per week. This exercise program was specifically developed for the present study and has not been previously evaluated or documented in other studies. One session was supervised by a kinesiologist (bachelor’s degree in kinesiology and doing her master’s in human kinetics) and was conducted via the Zoom platform (once a month, this session took place in person at the Université du Québec à Trois-Rivières) and two sessions were unsupervised and performed at home. All supervised and unsupervised sessions included a standardized 5-minute warm-up, followed by targeted exercises designed to strengthen the lumbo-pelvic-hip core musculature and enhance spinal and pelvic stabilization and alignment of the spine and pelvis (transversus abdominis, internal obliques, multifidus, pelvic floor, thigh, and hip muscles) [34, 35]. Although the exercises may appear simple, this approach was intentional to ensure accessibility for pregnant women with varying levels of physical conditioning, including those without prior exercise experience, and to accommodate the increased physical demands associated with pregnancy. All sessions ended with a 5-minute stretching. No specific equipment was needed to complete the program. The exercise program started ≤ 20 weeks of gestation and ended at 34–36 weeks of gestation. Several options for each exercise were offered in order to adapt the exercise program to the stage of pregnancy and related discomfort (see Fig. 1). Progressions and regressions were systematically proposed to maintain an appropriate level of challenge while prioritizing safety. At each supervised exercise session, the kinesiologist followed up on general and musculoskeletal health and, if needed, adapted the program, explained the new exercises and the goals of the changes made to the program. The objective was to ensure a safe and individualized program for each participant, and favor adherence. The kinesiologist recorded the number of exercise sessions completed per week, as well as any participant-reported modifications to the program, including changes in session frequency or the number of exercises performed.

#### Control group

Women randomized to the control group received standard prenatal care only, including general information on the management of PLBPP as outlined in the practical guide, *From Tiny Tot to Toddler* [33], but did not receive any structured exercise intervention during pregnancy. At 6–8 weeks postpartum, these participants were contacted to assess their needs for a tailored exercise program to manage PLBPP postpartum, ensuring that both groups had access to similar postnatal support.

### Data collection

#### Primary outcome measures

- The feasibility parameters included recruitment, retention and adherence rates as well as safety and acceptability of the intervention.

The recruitment rate was evaluated based on the number of eligible pregnant women recruited over a 12-month period, with successful recruitment defined as enrolling 40 women within this timeframe. The retention rate was determined by the completion of both the pre- and post intervention questionnaires, and a successful retention rate was defined as ≥ 80% of the recruited participants [17]. Adherence was defined as participation in both supervised and unsupervised exercise sessions. Completion of the unsupervised home-based sessions (including the warm-up, strengthening, and stretching components) was verified at each supervised visit and documented in the participant’s file by the kinesiologist. Successful adherence was defined as participation in ≥ 75% of all supervised and unsupervised exercise sessions [17]. Only adherence was evaluated and not fidelity since the latter was not part of our feasibility indicators.

The safety of the intervention was determined based on the number of adverse events mention by the participant, monitoring throughout the program. Adverse events were defined as symptom flare-ups preventing participation in subsequent exercise sessions or injuries requiring medical attention. At each supervised exercise session, a kinesiologist systematically asked participants about symptom exacerbation or injuries and recorded any adverse events in the women’s file.

Finally, the acceptability of the intervention (i.e., how the intervention was perceived by the participants) was assessed using 6-point Likert scales. The question asked was: “For the supervised and unsupervised exercise sessions, please rate on a scale from 0 to 5 your overall level of acceptability (i.e., choice, progression and intensity of the exercises, balance between supervised and unsupervised exercise sessions, mode of the supervised exercise sessions (via Zoom or in-person), considering that 5 represents the highest level of acceptability of the intervention. In addition, at the post-intervention visit, participants were invited to provide verbal feedback on what they liked or disliked about the exercise sessions.

#### Secondary outcome measures

- PLBPP frequency, and intensity; PLBPP-related disability.

At the pre- and post-intervention visits, current PLBPP intensity was evaluated by a visual analog scale (0 to 100 mm) and functional disability associated with PLBPP was measured using the Pelvic Girdle Questionnaire (PGQ) [23]. Throughout the intervention, participants in both groups received weekly text messages to collect information on PLBPP frequency (number of days) and intensity (average PLBPP intensity during the day and night over the past week) as well as any other treatments they received during the same period. Pain bothersomeness was not assessed through weekly text messages. The questions asked were: Please indicate the number of days you experienced PLBPP, 2) Please rate your average nocturnal and diurnal PLBPP intensity over the past week (using the same scale used at the pre-intervention visit, i.e. 0 (no pain) to 100 (maximum pain)) and 3) Please indicate whether you have received any treatment to manage PLBPP over the past week and, if so, each type of treatment and the number of sessions for each type of treatment. Participants reported this information in two parts: first, the type(s) of treatment received in the past week, and second, the number of sessions for each treatment type.

#### Additional questionnaires

Several variables potentially influencing the experience and functional impact of PLBPP were collected, including PA levels, fear-avoidance, anxiety, and depressive symptoms [36, 37]. Psychosocial factors may affect pain perception, coping, and functional limitations in musculoskeletal pain [36, 38, 39], while PA levels may relate to symptom severity and functional capacity during pregnancy [37]. These variables were measured at baseline to describe the population and ensure comparability between intervention and control groups, and reassessed post-intervention to contextualize the preliminary effects. Baseline comparisons were conducted to identify potential confounding differences.

PA levels were assessed with the Pregnancy Physical Activity Questionnaire (PPAQ). This questionnaire provides a comprehensive assessment of four PA domains, including “Sports and Exercises”, “Household and Caregiving”, “Transportation”, and “Occupation” [22]. The duration of time in each activity is multiplied by its intensity to arrive at a measure of average weekly energy expenditure (MET-h*week^− 1^) attributable to each activity [40]. Fear-avoidance behavior was assessed using the Tampa Scale of Kinesiophobia (TSK) [24], which evaluates the fear of movement and PA resulting from being afraid to get hurt [41]. Anxiety levels were assessed using the State-Trait Anxiety Inventory (STAI-Y) [25]. This questionnaire comprises two distinct scales: situational anxiety (current emotional state of the individual) and anxiety trait (emotion intensity). Finally, the Beck Depression Inventory (BDI) was used to evaluate different specific behavioural manifestations of depression [26]. More details about these questionnaires and scoring methods have been published elsewhere [17].

#### Additional information collected

At the pre-intervention visit, the following information was collected: age, gestational age, pre-pregnancy weight, height and educational level. Weight was also measured at the pre- and post-intervention visits to calculate gestational weight gain during the intervention, as it might be a confounding factor.

### Statistical analysis

Demographic and pre-intervention characteristics of the women, as well as feasibility data, are presented using means and standard deviations (SD), 95% confidence intervals (CI), and/or numbers and percentages. Normality of all data was verified by visual inspection of data distribution and the Kolmogorov-Smirnov test which provides graphical and numerical normality assessment. Student’s t-test for independent samples (for continuous variables), as well as the Pearson chi-square test (for categorical variables) were used to compare characteristics of the women from the intervention and control groups. Statistical significance was set at *p* < 0.05.

The preliminary effectiveness of the intervention on current PLBPP was assessed using descriptive statistics (means and 95% confidence intervals) to compare pre- and post-intervention changes between groups. For PLBPP frequency and nocturnal and diurnal pain intensity, collected weekly, the values from the first three weeks and the last three weeks of data collection were averaged to produce pre‑ and post‑intervention summary scores.

## Results

### Primary outcome measures

#### Participant flow and recruitment rate

**Fig. 2:**
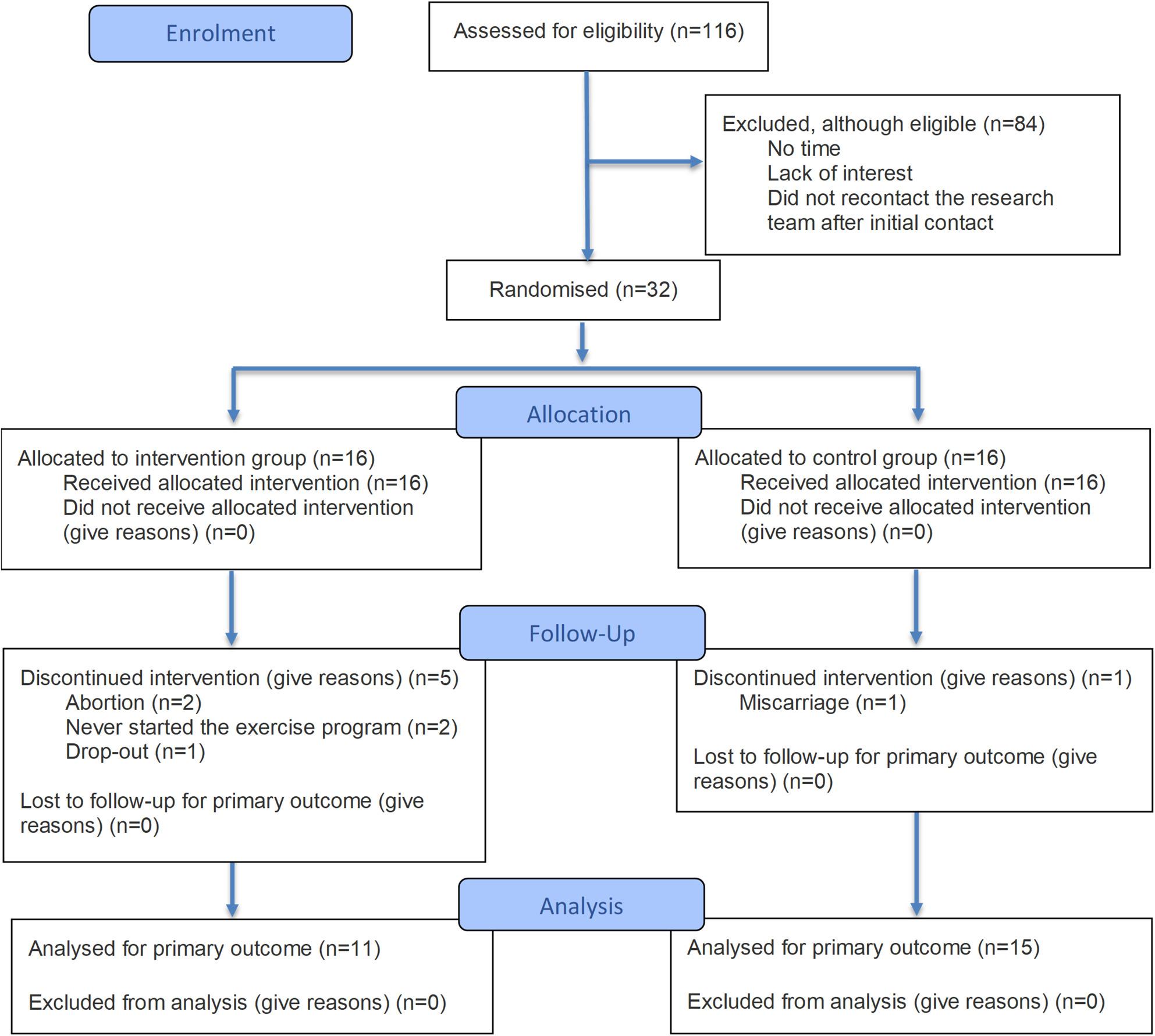
Flowchart of the pilot trial

Figure 2 presents the flow chart of the study. The study was presented to 116 eligible women, of whom 32 were recruited over a 15-month period. Our recruitment objective of 40 participants in 12 months was therefore not reached. The reason for not agreeing to participate was mostly associated with time commitment. Some women also reported a lack of interest in the project, while others requested time to consider participation but did not subsequently contact the research team.

#### Baseline data

Demographic and pre-intervention characteristics of the 32 women are presented in Table 1. Results showed a level of education that was higher in the control group, with more women holding a university degree (χ^2^ = 4.80, *p* = 0.03). Although not significant, parity tended to be higher in the intervention group (χ^2^ = 3.14, *p* = 0.08). At study inclusion, 27 participants presented with current PLBPP and five presented with a history of PLBPP, but no current PLBPP. PLBPP intensity was considered moderate [42]. No difference was found between the groups, and all variables were normally distributed. Since no significant baseline differences were observed between groups on additional questionnaires, they were not included as covariates in the analyses.

**Table 1:** Demographic and pre-intervention characteristics of the participants

	Intervention group (*N* = 16)	Control group (*N* = 16)	*p*
Pre-pregnancy characteristics			
Age (years)	29.9 [26.8–32.9]	30.9 [28.9–32.8]	0.56
History of LBP prior to current pregnancy			
Yes	12 (75.0%)	10 (62.5%)	0.45 †
No	4 (25.0%)	6 (37.5%)	
Parity			
0	5 (31.3%)	10 (62.5%)	0.08 †
≥ 1	11 (68.8%)	6 (37.5%)	
Education level			
Non-university degree	9 (56.3%)	3 (18.8%)	0.03 †
University degree	7 (43.8%)	13 (81.3%)	
Pre-pregnancy BMI (kg/m^2^)	24.6 [21.3–28.0]	24.7 [21.4–28.0]	0.98
Pre-pregnancy BMI categories			
< 25.0	10 (62.5%)	11 (68.8%)	0.88 †
25.0 to 29.9	3 (18.8%)	3 (18.8%)	
≥ 30.0	3 (18.8%)	2 (12.5%)	
Pre-intervention characteristics			
Current PLBPP			
Yes	13 (81.3%)	14 (87.5%)	0.66 †
No	3 (18.8%)	2 (12.5%)	
Current PLBPP intensity (/100)	24.1 [10.2–37.9]	29.5 [14.1–45.0]	0.58
PGQ	21.0 [13.3–28.7]	31.4 [23.3–39.5]	0.06
PPAQ	259.7 [191.6–327.8]	230.8 [189.9–271.7]	0.44
TSK	32.3 [28.8–35.7]	31.7 [28.1–35.3]	0.81
STAI-Y^a^	32.7 [27.3–38.1]	30.1 [25.9–34.3]	0.42
STAI-Y^b^	35.6 [29.5–41.6]	36.6 [31.8–41.4]	0.79
BDI	7.9 [4.9–10.9]	8.0 [5.7–10.3]	0.97
Current weight (kg)	67.8 [59.6–75.9]	70.2 [58.7–81.6]	0.72

CI, confidence intervals. Data are presented as mean [95% CI] or n (%). † chi-squared

BMI: body mass index; LBP: low back pain, PLBPP: pregnancy-related lumbopelvic pain; PGQ: Pelvic Girdle Questionnaire – score of disability (% disability); PPAQ: Pregnancy Physical Activity Questionnaire – Total activity (MET-h·wk^−1^); TSK: Tampa Scale of Kinesiophobia (/68); STAI-Y: State-Trait Anxiety Inventory - ^a^situational anxiety (/80), ^b^anxiety trait (/80); BDI: Beck Depression Inventory (/63)

#### Retention rate

Among participants randomized to the intervention group, two dropped out before the 1st exercise session and therefore never started the intervention. Two participants underwent medication-induced abortion after initiating the intervention; these events were unrelated to study participation and led to early discontinuation. One participant drop-out due to relocation (time constraints). In the control group, one participant experienced a miscarriage but had not participated in any study-related activities other than the text message follow-up.

Therefore, 26 participants completed the final post‑intervention questionnaires, resulting in an overall retention rate of 81.3%, which met our predefined criterion of ≥ 80% retention. When examined by groups, the control group achieved a retention rate of 94% (15/16), whereas the intervention group had a retention rate of 69% (11/16). Further details on the reasons for loss to follow‑up are presented in Fig. 2. Between May 19, 2021, and November 16, 2022, the intervention, through supervised training sessions, was delivered a total of 155 times, including 26 sessions conducted in person at UQTR and 129 sessions conducted remotely via Zoom.

#### Adherence rate

**Fig. 3:**
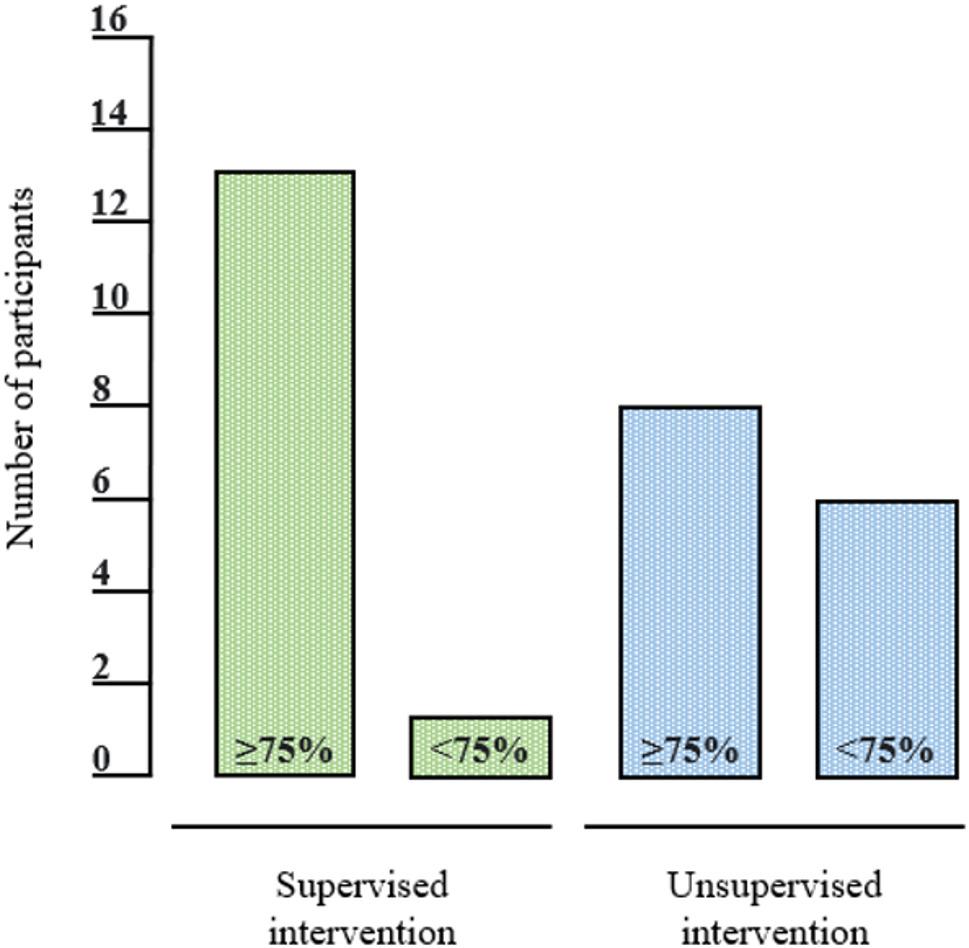
Adherence rates during supervised (green) and unsupervised (blue) exercise sessions

On average, the intervention lasted 14 weeks and the adherence rate is presented in Fig. 3. Of the 14 participants randomized to the intervention group and who started the intervention, 13 participants (92.9%) completed ≥ 75% of supervised exercise session and eight participants (57.1%) completed ≥ 75% of unsupervised exercise sessions and met our criteria for the supervised but not for the unsupervised exercise sessions.

#### Safety of the intervention

No participant reported adverse events over the course of the intervention.

#### Acceptability of the intervention

Eleven participants in the intervention group assessed the acceptability of the intervention which was higher for the supervised exercise sessions (4.59/5, 95% CI [4.3–4.9]) compared to unsupervised exercise sessions (3.98/5, 95% CI [3.7–4.3]), *p* < 0.001). Generally, participants described the supervised exercise sessions as more motivating, mainly because of the interaction with the kinesiologist and the possibility to immediately adjust the exercises if needed, either by correcting the movement or by changing the level of difficulty. On the other hand, unsupervised exercise sessions were appreciated because women could perform the program whenever they wished, without needing to inform a kinesiologist, as required during supervised sessions. However, they reported lower motivation to complete the program and sometimes shortened their sessions by reducing rest periods between exercises or omitting strengthening or stretching components.

### Secondary outcome measures

Results showed that changes from pre- and post-intervention for the secondary outcomes related to PLBPP, as well as for all additional outcomes, presented no clear differences between women from the intervention group and those from the control group (Table 2).

**Table 2:** Changes between pre- and post-intervention visit for each group

	Intervention group (*N* = 11)	Control group (*N* = 15)
Current PLBPP intensity (/100)	8.3 [− 6.3–22.8]	1.3 [− 11.5–14.1]
PGQ	15.9 [5.3–26.4]	12.4 [4.2–20.7]
Weight (kg)	7.3 [5.3-9.3]	8.4 [6.5–10.2]
PPAQ	− 37.2 [− 74.7–0.2]	− 17.9 [− 45.6–9.8]
TSK	0.8 [− 1.8–3.4]	1.8 [− 2.1–5.7]
STAI-Y^a^	0.1 [− 4.7–4.9]	1.8 [− 3.5–7.1]
STAI-Y^b^	− 3.0 [− 13.2–7.2]	− 1.7 [− 1.7−5.7]
BDI	-0.5 [− 4.5–3.6]	0.9 [− 2.5–4.4]

CI, confidence intervals. Data are presented as mean change [95% CI]

LBPP: lumbopelvic pain; PGQ: Pelvic Girdle Questionnaire – score of disability (% disability); PPAQ: Pregnancy Physical Activity Questionnaire – Total activity (MET-h·wk^− 1^); TSK: Tampa Scale of Kinesiophobia (/68); STAI-Y: State-Trait Anxiety Inventory - ^a^situational anxiety (/80), ^b^anxiety trait (/80); BDI: Beck Depression Inventory (/63).

When comparing weekly PLBPP frequency and nocturnal and diurnal intensity obtained during the first 3 and last 3 weeks of data collection between the groups (Table 3), no clear differences were observed.

**Table 3:** First and last three weeks of data collection for intervention and control group

	Intervention group		Control group	
	First 3 weeks (*N* = 14)	Last 3 weeks (*N* = 10)	First 3 weeks (*N* = 15)	Last 3 weeks (*N* = 14)
PLBPP frequency (/7)	6.1 [5.2–7.0]	5.6 [3.9–7.3]	5.71 [4.8–6.6]	5.6 [4.4–6.8]
Nocturnal PLBPP intensity (/100)	24.9 [9.2–40.7]	17.5 [2.9–32.2]	23.7 [12.0–35.5]	23.0 [10.6–35.3]
Diurnal PLBPP intensity (/100)	28.2 [16.0–40.5]	21.4 [10.8–31.9]	34.7 [26.0–43.5]	34.4 [22.3–46.5]

CI, confidence intervals; PLBPP, pregnancy-related lumbopelvic pain. Data are presented as mean [95% CI]. Data obtained in the first and last three weeks of data collection were averaged

A total of 15 women in the control group and 12 women in the intervention group provided data on treatments used outside the study to manage PLBPP. In the control group, 13 women reported using at least one treatment (total treatment used = 102) whereas in the intervention group, five women reported using at least one treatment (total treatment used = 41, *p* < 0.05). The interventions used by the participants (in no particular order) were chiropractic care, massage, physiotherapy, prenatal yoga class, osteopathy and other treatments. See Additional File 1 for more information.

## Discussion

In this pilot study, we found that implementing a motor control exercise program for pregnant women with a history of lumbopelvic pain or current PLBPP was feasible, although several feasibility indicators highlighted areas needing adjustments. Recruitment was lower than anticipated, with 32 participants enrolled compared to the targeted 40. Global retention met the predefined feasibility criterion (≥ 80%), whereas adherence met the criterion for supervised sessions but not for unsupervised home-based sessions. Importantly, no adverse events were reported in the intervention group, supporting the safety of the program. Overall, these findings suggest that conducting a subsequent pilot study rather than a full-scale trial would be important. A closer collaboration with prenatal care providers and diversified recruitment strategies (e.g., referral by clinicians and early pregnancy education settings) could be implemented to reach a broader and more diverse population of pregnant women. Retention and adherence could be improved through flexible intervention delivery and enhanced participant engagement. Retention may be supported by flexible scheduling, reduced in-person visits, and ongoing communication with participants, while adherence, particularly to home-based sessions, could be enhanced by increasing supervised sessions or by using hybrid approaches (e.g., virtual group sessions or real-time feedback), which were reported as more motivating. Given the pilot nature of this study, implications for planning a future adequately powered trial should be interpreted cautiously.

### Feasibility of the intervention

The Covid-19 pandemic likely affected our capacity to attain our recruitment objective of 40 participants in a 12-month period. Although recruitment occurred during the Covid‑19 pandemic (April 2021 to July 2022), this pilot study took place after the most acute phase of the health crisis, when restrictions had evolved and differed across countries, and virtual communication tools were increasingly integrated into routine prenatal care. This context may have influenced participants’ familiarity with and acceptance of remote delivery formats. The Covid-19 pandemic has affected the entire population, including pregnant women who would frequently leave their homes for their pregnancy follow-ups. A study of 2740 pregnant women from 47 states in the United States showed that 25.5% of the women stopped their in-person pregnancy visits when Covid-19 began, while some women chose to do their visits by video (15.2%) or by telephone (31.8%) [43]. As one of our recruitment methods was to meet women during their in-person pregnancy follow-ups to present them with our project, we likely met fewer women due to the pandemic. Moreover, although we adapted our protocol to limit face-to-face visits, by replacing in-person group exercise sessions with virtual individual exercise sessions using the Zoom platform, it is nevertheless possible that pregnant women were reluctant to participate in a project requiring face-to-face visits (i.e. pre- and post-intervention visits) outside of the environments they are used to dealing with. Moreover, the use of electronic equipment and new technology associated with the use of the Zoom platform, which requires a certain level of ease, and the delivery a virtual exercise intervention, which might have raised concern about the safety of the program, and may have negatively affected recruitment. Before taking part in a research project, pregnant women tend to seek information about the various elements that characterize the project, in order to make an informed decision about their participation. As a result, the availability of information and research awareness from the research team and clinicians to the pregnant women seems to play a key role in a woman’s decision whether or not to participate in a study, and thus, advancing specific knowledge about this unique population of women and PLBPP management [44]. Although pregnant women, clinicians and researchers share certain concerns about the risks and benefits of research, women seem to place greater importance on protecting themselves and their fetus, while clinicians are more concerned with meeting regulatory requirements for the ethical conduct of the research, and researchers are concerned with balancing the benefits and risks of the research project [44]. Since some women are better equipped to deal with the problems that arise during pregnancy, it becomes important to adapt the research approaches chosen to recruit participants. Therefore, it is important to acknowledge the specific needs and characteristics of pregnant women, as well as the ethical and financial constraints that may exist across settings, to reach diverse populations of women and thus obtain more widely generalizable results.

Individuals with lumbopelvic pain in a general non-pregnant population often seek herbal or natural health products as well as manual therapy approaches such as chiropractic care and massage therapy [45]. In our pilot study, none of the participants reported using herbal or natural health products, which may reflect greater caution around these products during pregnancy. Also, most participants (especially in the control group) had completed university-level education, which may have increased their awareness of available pain‑management treatments.

Overall retention rates were very good (81.3%) but was lower in the intervention group compared to the control group. However, when excluding the two women that experienced pregnancy-related events unrelated to the intervention (abortion), retention rates between the groups would have been similar, suggesting that such pregnancy-related events may influence retention independently of the intervention.

Whereas adherence to the supervised exercise sessions was excellent, adherence to the unsupervised exercise session was much lower, with only 57% of women having completed ≥ 75% of the sessions. Based on the participant’s feedback at the end of the intervention (acceptability of the intervention), unsupervised sessions were considered less motivating because they had to perform them independently, without the real-time supervision and support of the kinesiologist that was available during the supervised sessions. The women in the intervention group reported no adverse events (safety of the intervention) that prevented them from carrying out their tasks or activities of daily living. As they were accompanied individually by a kinesiologist each week during the supervised intervention, they reported feeling confident and comfortable mentioning to the kinesiologist if an exercise no longer suited them because of discomfort. This might have prevented potential adverse events. Among the three participants who did not complete the intervention, two discontinued due to medication-induced abortions (unrelated to the study), while only one dropout was related to feasibility (relocation). As such, dropout attributable to intervention or feasibility factors was low, and adherence among participants who complete the intervention suggests that the prescribed frequency of three 40-minute sessions per week was generally well tolerated. Although most women in the intervention group had one or more children, the potential impact of childcare demands on adherence remains an interpretation, as participants did not explicitly report this as a barrier. Other factors related to remote delivery, such as occasional technical issues or lower motivation in the absence of live interaction, may also have contributed to reduced adherence to the unsupervised sessions. Strategies to increase adherence may include providing more supervised exercise sessions, as these were perceived as more motivating (better acceptability than the unsupervised sessions), or implementing approaches to enhance motivation for unsupervised sessions, such as fostering a sense of belonging to the project or creating friendly competition among participants.

### Implications for a transition to a full-scale trial

To move on to a full-scale study, recruitment of women with a higher level of baseline PLBPP intensity and associated disability would increase the likelihood of detecting a significant effect on the motor control exercise program. Moreover, modifying minimization criteria, by adding education level and parity, but removing baseline PA levels, would prevent the risk of imbalance between groups for these potential confounding factors.

Although preliminary evidence regarding the effectiveness of the motor control exercise program on LBPP intensity remains limited, exercise is considered one of the most recommended interventions for managing PLBPP. Indeed, a recent systematic review and meta-analysis including 22 articles showed that exercise, compared to control, was effective (overall ES = 2.07; 95% CI = 1.35–2.78; *P* < 0.001) to reduce lumbopelvic pain intensity during pregnancy [46]. However, this review also highlighted important limitations, including the inclusion of studies with a high risk of bias, potential publication bias, substantial heterogeneity, and a lack of clarity and consistency in the reported interventions, which limits the strength and interpretability of the findings and justifies further research. One possible explanation for the discrepancy is that this pilot study was not powered to detect between-group differences and adherence to unsupervised sessions was lower than expected.

### Strengths and limitations

Our randomized controlled pilot trial has strengths and limitations that should be discussed. Among the strengths, we developed a motor control exercise program that was tailored to the gestational stage (e.g., trimester of pregnancy) and related PLBPP discomfort, as well as the level of experience (or PA habits) of the women. To evaluate an intervention targeting PLBPP, we included pregnant women with a history of lumbopelvic pain or who are currently experiencing PLBPP, as they constitute the target population for the program. A history of LBP and PGP is a known risk factor in the development of pain in the lumbopelvic area during pregnancy [3]. It was essential to include not only women currently experiencing pain, but also those with a history of LBP and/or PGP. Due to the Covid-19 pandemic, we had to adapt our protocol and included telerehabilitation (i.e., means of delivering rehabilitation interventions through digital technologies and communication). While the need to use electronic equipment and potential safety concerns about safety in a virtual program may have negatively affected recruitment, the convenience and scheduling flexibility of Zoom-based supervised sessions may have positively supported retention and adherence. This mode of delivery should be considered in a future pilot trial. Limitations of our study include the lack of blinding of the individuals who conducted the pre- and post-intervention outcomes assessment. For feasibility reasons, including limited human and financial resources, we could not conduct a full double-blinded trial (participants and outcome assessor), but this should be implemented in future trials. The high proportion of eligible women who declined participation highlights important recruitment challenges. The limited and non‑systematic documentation of reasons for non‑participation is a limitation of this study and should be addressed in future trials, ideally through patient and public involvement to better align study demands with participant expectations and constraints. The acceptability was assessed only for the exercise intervention and not for the overall participation in the trial, including the time commitment required. This information would have been important to inform the planning of another pilot study as time needed to participate in a trial, and specifically when randomized to the intervention group, influence recruitment, adherence and retention rate, and therefore the effectiveness of the intervention. This study did not include patient and public involvement at any stage of its planning or design. The inclusion of a patient partner could have informed key aspects of the intervention, such as supervised and unsupervised sessions, as participants later reported greater appreciation for supervised training with a kinesiologist. Future studies should integrate patient and public involvement to improve intervention relevance and acceptability. Furthermore, the small non-significant difference observed between groups may partly reflect attention bias, as participants in the intervention group received more frequent interaction with a kinesiologist. This should be addressed in another pilot study by considering attention matched control conditions. Another limitation is the participant presents relatively low baseline intensity of current PLBPP in both groups, with mean values below levels often considered clinically meaningful or below the patient acceptable symptom state (PASS) in musculoskeletal pain conditions in a non-pregnant population [47]. This may have reduced women’s perceived need for intervention, potentially influencing both their willingness to enroll in the study. It should be noted that the validity of some of the clinical tests (Trendelenburg and Patrick’s FABERE test) used to support the diagnosis of PLBPP has not yet been specifically established, which may limit the strength of the diagnostic inferences drawn from these assessments. Finally, the overall over-representation of highly educated pregnant women suggests a possible selection bias and a limited external validity of our study. In a future trial, efforts should be made to raise awareness among less educated women to improve sample diversity. In addition, outreach strategies, design-based approaches, such as stratified randomization based on education level, as well as analytic approaches if imbalances persist (e.g., adjustment for education level), could also improve representativeness and strengthen internal validity in a future pilot study. However, given that several feasibility criteria were not fully met, a full-scale RCT is not currently planned. Further pilot study is needed to optimize study procedures, such as recruitment strategies and adherence to the intervention, before considering a definitive trial.

## Conclusion

Our data suggests that the motor control exercise program we tested in this pilot study among pregnant women with a history of, or currently suffering from PLBPP, is safe and feasible with some minor adjustments. Attention should be given to recruitment strategies and adherence before progressing to a full-scale randomized controlled trial.

## Supplementary Information

Below is the link to the electronic supplementary material.

Supplementary Material 1

Supplementary Material 2

Supplementary Material 3

## Data Availability

The datasets used and/or analysed for the current study is available from the corresponding author on reasonable request.

## Abbreviations

BDI: Beck depression inventory
BMI: Body mass index
CIUSSS-MCQ: Centre Intégré Universitaire de Santé et de Services Sociaux de la Mauricie-et-du-Centre-du-Québec
CI: Confidence intervals
LBP: Low back pain
PA: Physical activity
PGP: Pelvic girdle pain
PGQ: Pelvic girdle questionnaire
PLBPP: Pregnancy-related lumbopelvic pain
PASS: Patient acceptable symptom state
PPAQ: Pregnancy physical activity questionnaire
STAI-Y: State-trait anxiety inventory
TSK: Tampa scale questionnaire
