# Supplementary material for: Motor control exercise program versus standard care in the treatment of lumbopelvic pain in pregnant women: a randomized controlled pilot trial
Source: Chiropr Man Therap. 2026 May 30;34:30. doi: 10.1186/s12998-026-00655-x (PMC13430806; doi:10.1186/s12998-026-00655-x)
Supplement: Supplementary file 1 — Supplementary Material 1 [file 12998_2026_655_MOESM1_ESM.docx]

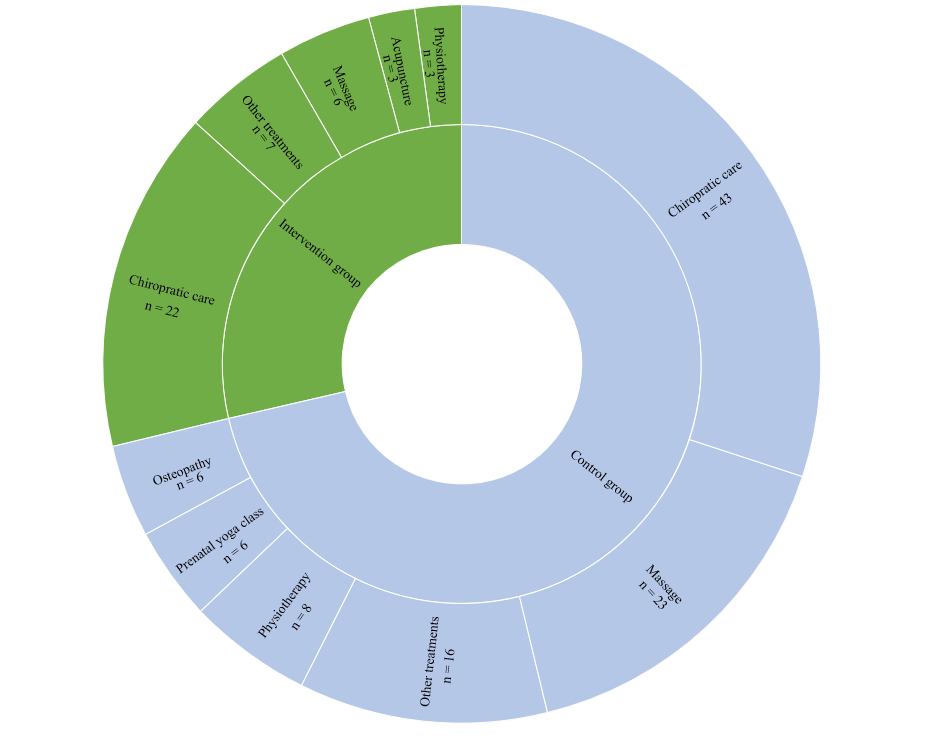


Additional file 1

Title: Proportion of treatment received by participants outside of the study.

Description: The intervention group is shown in green, and the control group is shown in blue. "N" presented refers to the number of treatment sessions received by the participants.
